# Researches on the Ovum and Corpus Luteum of Man and the Lower Animals

**Published:** 1845-04

**Authors:** 


*Researches on the Ovum and Corpus Luteum of Man and the lower Animals.* By M. Deschamps.—In our Number for April, 1844, we gave an account of the interesting researches of M. Raciborski on the periodical deposition of ova by woman and the females of the lower animals, and as M. Deschamps' views, though not apparently founded on such extensive inquiries, differ in one important point from those of M. Raciborski, we give a short extract of them.

M. Raciborski states that during the menstrual or rutting season one or more ova are developed and thrown off, whether the ova be impregnated or not, and that the place from which the ovum or ova are thrown off is marked by a *corpus luteum.* M. Deschamps denies this, and states that a corpus luteum is alone formed when impregnation has taken place, and that the periodic deposition of ova during the menstrual or rutting season is only followed by a kind of false cicatrix, but by no corpus luteum. While M. Raciborski therefore regards the existence of a corpus luteum as simply marking the menstrual periods, and proves it by a reference to numerous dissections, M. Deschamps regards it as a "certain indication of the fruitful union of the sexes," but refers to no dissections in point. M. Deschamps' description of the true corpus luteum exactly agrees with that which M. Raciborski describes in many species of animals as occurring every menstrual period without access to the male. M. Deschamps describes the ovum at every menstrual period not as being deposited, but as bursting or shrivelling, unless impregnation takes place, when he conceives it is expelled by a particular mechanism. M. Raciborski, on the other hand, from a reference to his numerous dissections of the ovaries of birds and the lower animals, shows that the ovum is discharged entire at every menstrual period, and by the same mechanism as when impregnation has taken place; that the deposition of the ovum and the formation of a corpus luteum are quite independent of access to the male; and that in some entire classes of animals the ovum is not fructified till it be expelled from the body of the fe male, or is just ready to be so.—*Ed. Med. and Surg. Jour., from Gaz. Med. de Paris.*

